# Evaluating reliability of automated quantitative brain morphometry from fetal T2-weighted MRI

**DOI:** 10.3389/fnins.2026.1817732

**Published:** 2026-06-01

**Authors:** Seungyoon Jeong, Andrea Gondova, Hyuk Jin Yun, Sungmin You, Melquisideth Lagunas, Polina Golland, P. Ellen Grant, Kiho Im

**Affiliations:** 1 Fetal-Neonatal Neuroimaging and Developmental Science Center, Boston Children’s Hospital, Harvard Medical School, Boston, MA, United States; 2 Division of Newborn Medicine, Boston Children’s Hospital, Harvard Medical School, Boston, MA, United States; 3 Department of Pediatrics, Harvard Medical School, Boston, MA, United States; 4 Computer Science and Artificial Intelligence Laboratory, Massachusetts Institute of Technology, Cambridge, MA, United States; 5 Department of Radiology, Harvard Medical School, Boston, MA, United States

**Keywords:** cortical morphometry, fetal brain MRI, intraclass correlation coefficient, measurement reliability, NeSVoR, slice-to-volume reconstruction

## Abstract

**Introduction:**

Three-dimensional assessment of fetal cortical morphology from MRI is essential for understanding early brain neurodevelopment. However, measurement can be affected by fetal imaging quality, number and selection of available stacks, and reconstruction methods.

**Methods:**

We evaluated the within-session reliability of an automated cortical morphometry pipeline in 30 typically developing fetuses [22–36 weeks gestational age (GA)]. For each subject, two disjoint subsets of 2D T2-weighted stacks (no shared stacks) were independently reconstructed into 3D volumes using the Neural Slice-to-Volume Reconstruction (NeSVoR) and the Slice-to-Volume Reconstruction Toolkit (SVRTK). Cortical plate volume, surface area, mean sulcal depth, and absolute mean curvature were extracted, and measurement reliability was assessed using absolute percent difference (APD) and intraclass correlation coefficients (ICC). Multiple linear regression evaluated the effects of mean stack quality, quality difference between subsets, stack count, and GA on measurement reliability.

**Results:**

NeSVoR-derived metrics showed high reliability for all measures (mean APD < 3%, ICC > 0.99). SVRTK-derived metrics were also robust (mean APD < 5%, ICC > 0.97). Reliability increased with greater stack count and older GA in NeSVoR, and with higher mean stack quality in SVRTK.

**Discussion:**

These results demonstrate that automated cortical morphometry from fetal MRI yields highly consistent measurements of volumetric and surface metrics within the proposed within-session design, once minimum levels of image quality and stack count are met. This study proposes a within-session benchmark for automated fetal cortical measurements and underscores that systematic reliability assessment is essential for confident application of automated pipelines in fetal neuroimaging.

## Introduction

Fetal brain development plays a critical role in shaping long-term neurodevelopmental outcomes, highlighting the importance of accurate in utero assessment (Glenn, 2010; Rees and Inder, 2005; van den Heuvel and Thomason, 2016). In recent decades, fetal magnetic resonance imaging (MRI) has emerged as a powerful non-invasive modality for detecting subtle and complex prenatal brain abnormalities, offering superior soft-tissue contrast and spatial resolution compared to ultrasound (Griffiths et al., 2017; Manganaro et al., 2017; Moltoni et al., 2021). Additionally, quantitative fetal MRI enables characterization of neurodevelopmental processes through three-dimensional (3D) measures of brain morphology. While conventional 2D biometry remains clinically useful, 3D volumetric and surface-based metrics, such as whole-brain and regional cortical plate volume, cortical surface area, sulcal depth and mean curvature, provide a more comprehensive description of early brain growth and folding (Ortinau et al., 2019; Yun et al., 2021).

Previous works have demonstrated the relevance of these metrics, including rapid prenatal expansion of cortical plate volume (Andescavage et al., 2017), strong associations between mean brain curvature and gestational age (GA) (Wu et al., 2015), hemispheric asymmetries and sulcal maturation (Habas et al., 2012; Yun et al., 2022), and regional surface area expansion during fetal development (Wu et al., 2021). Such 3D quantitative measures may offer increased sensitivity for detecting atypical neurodevelopment (Im et al., 2017). However, their meaningful application critically depends on the reliability of the automated pipelines used to derive them.

Accurate quantification from fetal MRI remains challenging due to fetal motion, rapidly evolving anatomy, low effective resolution, and limited tissue contrast (Gholipour et al., 2010; Manganaro et al., 2017; Prayer et al., 2017). Motion-tolerant 2D acquisition sequences such as Half-Fourier Acquisition Single-Shot Turbo Spin Echo (HASTE; Yamashita et al., 1997) reduce intra-slice motion but do not mitigate inter-slice misalignment, resulting in disrupted 3D spatial coherence (Im, 2021). To address this, fetal imaging protocols typically acquire multiple stacks in different orientations (Glenn, 2010), which are subsequently integrated using slice-to-volume reconstruction (SVR) algorithms to generate motion-corrected 3D volume (Gholipour et al., 2010; Kuklisova-Murgasova et al., 2012; Uus et al., 2020; Xu et al., 2023). The reliability of downstream morphometric measurements is therefore influenced not only by fetal anatomy, but also by acquisition quality, stack count that impact reconstruction methodology, as well as subsequent segmentation and surface extraction steps.

Despite the growing use of automated fetal brain morphometry, systematic evaluations of measurement reliability remain limited. Prior studies have primarily focused on the reliability of linear biometric measures (e.g., biparietal diameter, fronto-occipital diameter, or corpus callosum length) or segmentation overlap metrics (Ciceri et al., 2023; Specktor-Fadida et al., 2024). In contrast, the within-session reliability of whole-brain cortical plate volumetric and surface-based metrics; particularly under realistic variability in stack selection, quality, and count; has not been comprehensively assessed. Importantly, classical test-retest reliability paradigms are poorly suited to fetal neuroimaging. Even short inter-scan intervals of a few days can introduce substantial true biological changes in cortical volume, folding, and curvature, particularly during mid-gestation when growth rates are rapid. Repeated scans within the same day are often impractical and, when feasible, still might be considered a single imaging session. As a result, assessing reliability in fetal MRI requires alternative strategies that isolate acquisition- and reconstruction-related variability from genuine neurodevelopmental change.

In this study, we adopted a within-session split-sample framework to evaluate the reliability of automated fetal cortical morphometry. Disjoint subsets of 2D T2-weighted stacks acquired during a single imaging session were independently reconstructed into 3D volumes, enabling assessment of internal consistency under realistic acquisition variability. We examined how key imaging factors, including stack quality and stack count, as well as gestational age (GA), influence the stability of cortical plate volume and surface-based metrics. Neural Slice-to-Volume Reconstruction (NeSVoR) (Xu et al., 2023) was used as the primary reconstruction method, with the Slice-to-Volume Reconstruction Toolkit (SVRTK) (Uus et al., 2020) serving as a complementary reference, representing distinct and widely used SVR frameworks.

By quantifying within-session measurement variability across reconstruction methods and imaging conditions, this work establishes practical reliability benchmarks for automated fetal cortical morphometry. These benchmarks provide essential context for interpreting quantitative fetal MRI measurements in research settings and inform future methodological and clinical validation studies, while explicitly distinguishing technical reproducibility from biological or diagnostic validity.

## Materials and methods

### Subjects

This study included 30 typically developing (TD) singleton fetuses (GA: 29.89 ± 3.56 weeks [mean ± standard deviation (SD)], range: 22.00–35.57 weeks; sex: 21/8/1 [male/female/unknown]). TD fetuses were retrospectively selected from prior fetal research studies conducted at Boston Children’s Hospital (BCH) between 2014 and 2024, under IRB approval (IRB-P00008836 and IRB-P00040121), with parental consent obtained in accordance with institutional guidelines. Fetuses with dysmorphic features on ultrasound examination, known chromosomal abnormalities, known congenital infections, or any clinically significant brain abnormality on visual inspection of the fetal MRI were excluded.

### Data acquisition

MRI was acquired on a Siemens 3T Skyra scanner and included repeated multi-planar T2-weighted HASTE sequences with 1-mm in-plane resolution, field of view (FOV) = 256–320 mm, repetition time (TR) = 1,400–2,000 ms, echo time (TE) = 100–120 ms, and slice thickness = 2–4 mm. After localization of the fetal brain, a total of 7–31 HASTE stacks were acquired in three approximately orthogonal orientations (axial, sagittal, and coronal planes).

### Data organization for within-session design

**FIGURE 1:**
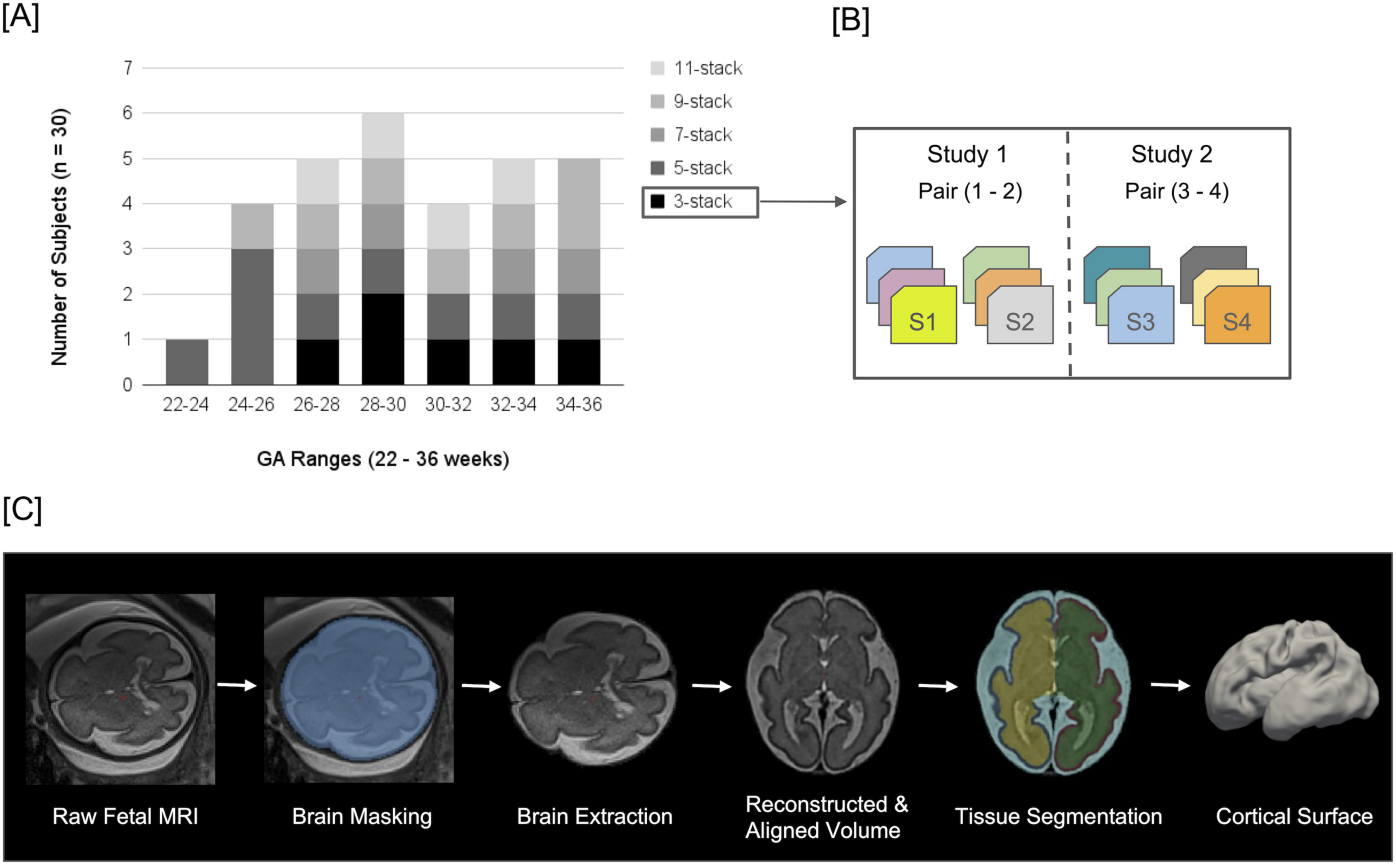
Study design, imaging subsets, and processing pipeline overview for within-session reliability analysis. **(A)** Histogram showing the distribution of 30 subjects across GA bins (22–36 weeks). Bars are colored according to the number of HASTE stacks selected for each subject (3, 5, 7, 9, or 11 stacks). **(B)** Diagram of the subset pairing process: for each subject, stacks were manually assigned to two separate subsets (S1–S2 for Study 1, S3–S4 for Study 2) for within-session comparisons. Each color-coded folder icon denotes an individual HASTE stack included in the corresponding subset. **(C)** Summary of the fetal brain MRI processing pipeline.

To assess the within-session reliability of automated brain metric extraction from fetal MRI, we performed two separate within-subject comparisons. In Study 1, multiple stacks in each fetus were manually split into two disjoint subsets (Subsets 1 and 2). A split-sample design using disjoint stack subsets was adopted to quantify measurement stability under realistic fetal MRI acquisition variability. To reduce dependence on a single random split, we performed the same task independently and generated a different pair of subsets (Subsets 3 and 4) in Study 2. Some stacks could be randomly reused across studies, but within each study, the paired subsets were mutually exclusive. This two-split design broadened the distribution of mean stack quality across subsets and reduced dependence on a single random split of stacks. Each pair was assigned to one of five predetermined stack-count conditions (3–3, 5–5, 7–7, 9–9, or 11–11 stacks per subset). Importantly, this design does not represent true test-retest but instead evaluates internal consistency under realistic within-session acquisition variability. Variation in stack count and quality within subsets allows assessment of their influence on measurement reliability. Figures 1A,B summarizes both the distribution of subjects across stack-count conditions and the assignment of imaging subsets for Studies 1 and 2. No systematic relationship between GA and stack count was observed upon visual inspection.

Next, each HASTE stack was assigned a quality score using a previously trained and validated machine learning model, which outputs a single continuous value reflecting the degree of motion distortion and overall stack quality. The model was originally trained on quality annotations provided by two fetal MRI experts on a five-point scale (1: very poor quality, unusable for biometric analysis due to severe motion and blurring; 2: poor quality, usable only for basic reconstruction with evident artifacts; 3: acceptable quality, suitable for standard reconstruction with some blurring but preserved anatomical detail; 4: good quality, minimal blurring and clear tissue boundaries; 5: excellent quality, sharp anatomical detail and negligible motion artifacts) (Supplementary Figure 1). In the present study, the machine learning model output was used directly as a single stack-level quality score. Stacks scoring 1 were excluded to maintain a realistic but analyzable range of image quality. A total of 3 stacks received a quality score of 1 and were excluded across the cohort. For each subset (across Studies 1 and 2), the mean quality score across its stacks was then calculated. For each pair, both the mean stack quality (across the two subsets) and the absolute difference in mean quality between subsets (“quality difference”) were computed, and included as independent variables in the subsequent regression analyses to evaluate their impact on within-session reliability of quantitative brain morphometry.

In addition to the primary imaging factors (stack quality, quality difference, stack count, and GA), slice-thickness variables (mean slice thickness and inter-subset thickness differences) were explored as secondary contributors to measurement reliability; results are reported in Supplementary material.

### Fetal MRI processing and inner cortical plate surface reconstruction

Fetal MRI processing and cortical plate surface reconstruction were performed using a previously established pipeline (You et al., 2024; Yun et al., 2021; Figure 1C) that included brain extraction (Hong et al., 2021) and N4 bias field correction (Tustison et al., 2010). Subsets of stacks were then combined using two different SVR methods to generate motion-corrected 3D volumes at 0.5 mm isotropic resolution. Our primary reconstruction approach was NeSVoR, a resolution-agnostic SVR algorithm that models the underlying volume as a continuous function of spatial coordinates using an implicit neural representation (Xu et al., 2023). To examine the robustness of morphometric measurements with respect to reconstruction variability, we also applied SVRTK, an iterative super-resolution framework that performs rigid-body alignment of slices to remove motion to reconstruct volumes (Uus et al., 2020). Both methods used identical preprocessed stack subsets to ensure consistency.

Cortical plate segmentation was performed using a deep learning-based method (Hong et al., 2020). Left and right hemispheric triangular surface meshes of the inner cortical plate were automatically extracted using a topology-preserving marching cubes algorithm (Lepage et al., 2017).

### Whole-brain cortical plate volume and surface measures

We quantified whole-brain cortical plate volume and surface-based metrics, including the area of the inner cortical plate surface (hereafter referred to as surface area), mean sulcal depth, and absolute mean curvature (hereafter referred to as mean curvature). Cortical plate volume was calculated as the number of cortical plate voxels multiplied by the voxel volume. Surface area was computed as the sum of the areas of the mesh triangles on the left and right inner cortical plate surfaces. Sulcal depth was measured as the shortest path from each vertex to the cortical plate convex hull using an adaptive distance transform method (Lepage et al., 2017; Yun et al., 2013), and mean sulcal depth was averaged across left and right surfaces. Mean curvature was estimated as the average of local angular deviations across all vertices (Meyer et al., 2003), using vertex-wise absolute values. This sign-invariant index has been widely used as a quantitative measure for cortical shape complexity (Luders et al., 2006; Yun et al., 2024).

To assess measurement reliability, metrics were plotted against GA to confirm expected developmental trends and identify potential outliers. No manual corrections were applied, but substantially deviant points were investigated to evaluate variability inherent to automated processing.

### Statistical analysis

Reliability of quantitative cortical measurements was first evaluated using the absolute percent difference (APD) between each pair of subsets (Subsets 1 vs. 2; Subsets 3 vs. 4), calculated as:


$$\mathrm{APD}=\left(\frac{\left|M_{1}-M_{2}\right|}{\frac{M_{1}+M_{2}}{2}}\right)\times 100\%$$

where M_1_ and M_2_ are measurements from the same fetus. Measurement consistency and agreement were assessed with intraclass correlation coefficients (ICCs) using a two-way random-effects model [ICC(2,1)], treating both subjects and within-session pairs as random factors (Koo and Li, 2016). Additionally, we performed Bland–Altman analyses to assess absolute agreement between paired measurements, deriving repeatability coefficients (RC) defined as half the width of the 95% limits of agreement, providing a measure of absolute reconstruction-induced variability in the original units of each metric. RCs are reported with 95% confidence intervals estimated via nonparametric bootstrap resampling (1,000 iterations at the subject level).

To investigate factors influencing reliability, namely: mean stack quality (mean quality across all stacks within each within-session pair), quality difference (absolute difference in mean quality between subsets), stack count, and GA, multiple linear regressions were performed (independently for measures obtained in Study 1 and Study 2) with APD as the dependent variable. Because fetus-level APD values were computed from two disjoint within-session stack subsets, the unit of analysis was the fetus, and no repeated dependent measurements per subject were included in the model. Model assumptions (linearity, homoscedasticity, and normality of residuals) were verified, and false discovery rate (FDR) correction was applied across the four cortical metrics within each study to account for multiple tests.

To assess robustness of regression estimates, a bootstrap resampling analysis was performed (1,000 subject-level iterations). In each iteration, subjects were resampled with replacement and the full regression model was refitted. Stability of effects was quantified using directional consistency, defined as the proportion of bootstrap iterations in which each regression coefficient retained the same sign as in the full-sample model.

All analyses were performed independently for NeSVoR and SVRTK reconstructions using MATLAB R2021b (The MathWorks Inc, 2021).

## Results

### Reliability of quantitative cortical measurements

**FIGURE 2:**
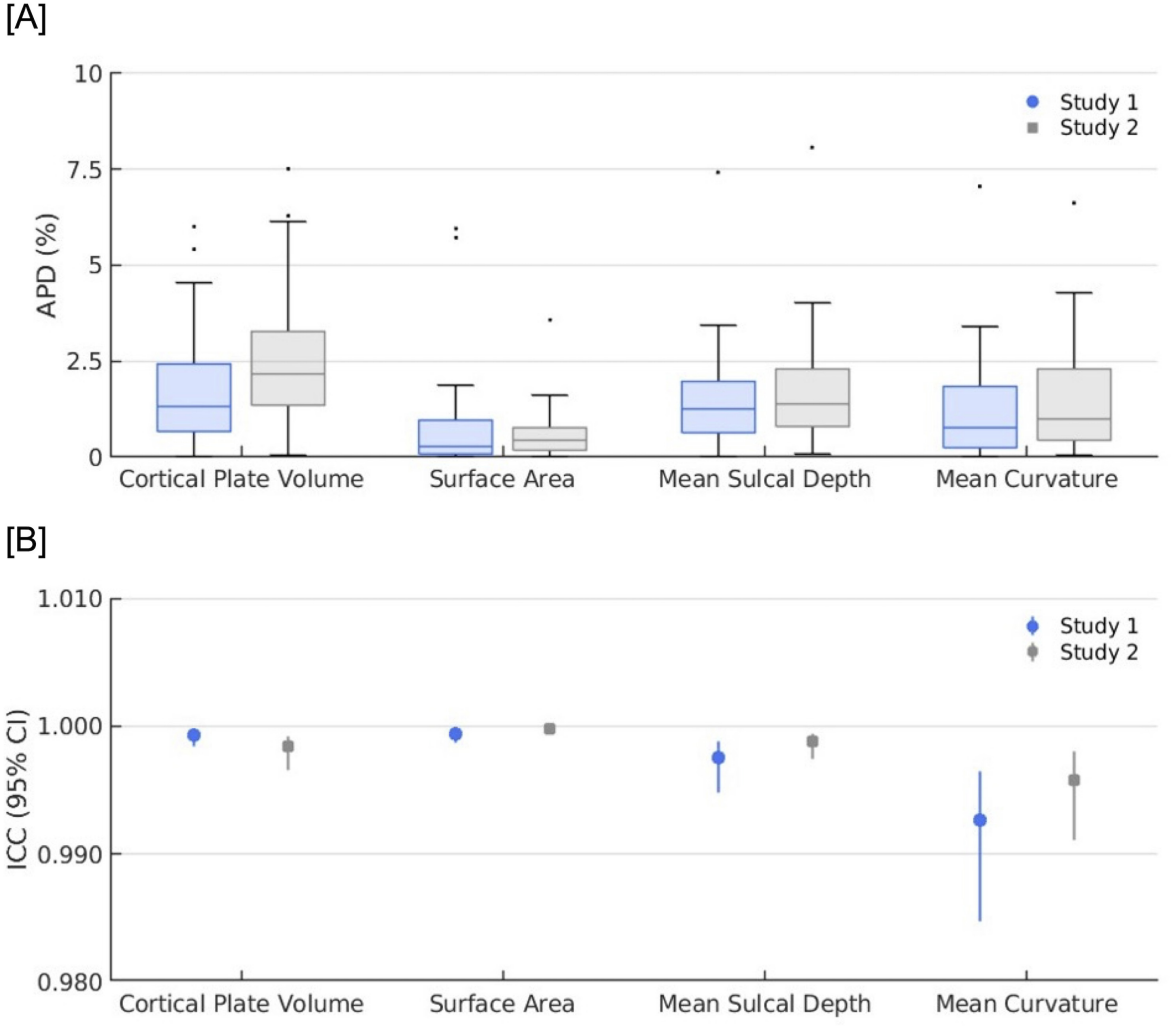
Within-session reliability of NeSVoR-derived cortical metrics. **(A)** Box plots display APD (%) across subjects for cortical plate volume, surface area, mean sulcal depth, and mean curvature in Study 1 and Study 2. **(B)** ICC(2,1) estimates with error bars indicating 95% CIs for cortical plate volume, surface area, mean sulcal depth, and mean curvature in Study 1 and Study 2 (dashed line at ICC = 1).

Automated extraction of fetal cortical metrics from NeSVoR reconstructions showed high within-session reliability, with low mean APD values and high ICCs (Figure 2 and Table 1). Cortical plate volume had mean APD of 1.78 ± 1.53% in Study 1 and 2.52 ± 1.85% in Study 2. Surface area also showed high within-session reliability (mean APD of 0.82 ± 1.45% and 0.60 ± 0.70% in Studies 1 and 2, respectively). Mean sulcal depth and mean curvature exhibited slightly higher mean APDs, ranging from 1.69 to 2.00% across both studies. ICC values were consistently high, ranging from 0.993 to 1.000 in Study 1 and from 0.996 to 1.000 in Study 2, indicating strong reliability across all metrics. RCs indicating within-session variability in the original measurement units were 1.93 cm^3^ [1.92, 3.31] in Study 1 and 2.81 cm^3^ [2.88, 4.91] in Study 2 for cortical plate volume, 765.2 mm^2^ [426.8, 1583.5] and 449.8 mm^2^ [415.5, 816.1] for surface area, 0.20 mm [0.15, 0.38] and 0.14 mm [0.15, 0.24] for mean sulcal depth, and 0.02 [0.01, 0.05] and 0.02 [0.01, 0.03] for mean curvature (see Supplementary Figure 4 for visualization of Bland-Altman plots).

**TABLE 1:** Within-session reliability of NeSVoR-derived quantitative cortical measurements.

Biometric measure	Study 1			Study 2		
	Mean APD (%) ± SD	ICC (95% CI)	RC (95% CI)	Mean APD (%) ± SD	ICC (95% CI)	RC (95% CI)
Cortical plate volume	1.78 ± 1.53	0.999 (0.998, 0.999)	1.93 (1.92, 3.31)	2.52 ± 1.85	0.998 (0.997, 0.999)	2.81 (2.88, 4.91)
Surface area	0.82 ± 1.45	0.999 (0.999, 1.000)	765.2 (426.8, 1583.5)	0.60 ± 0.70	1.000 (0.999, 1.000)	449.8 (415.5, 816.1)
Mean sulcal depth	1.98 ± 2.74	0.998 (0.995, 0.999)	0.20 (0.15, 0.38)	1.69 ± 1.53	0.999 (0.997, 0.999)	0.14 (0.15, 0.24)
Mean curvature	2.00 ± 4.31	0.993 (0.985, 0.997)	0.02 (0.01, 0.05)	1.97 ± 3.24	0.996 (0.991, 0.998)	0.02 (0.01, 0.03)

Absolute percent difference (APD, mean ± SD), intraclass correlation coefficients [ICC(2,1), 95% CI], and repeatability coefficients [RC, 95% CI] for cortical plate volume, surface area, mean sulcal depth, and mean curvature in Study 1 (S1–S2) and Study 2 (S3–S4). RC values are reported in cm^3^ for cortical plate volume, mm^2^ for surface area, mm for mean sulcal depth, and curvature units for mean curvature.

### Effect of mean stack quality, quality difference, stack count, and GA on measurement reliability

**FIGURE 3:**
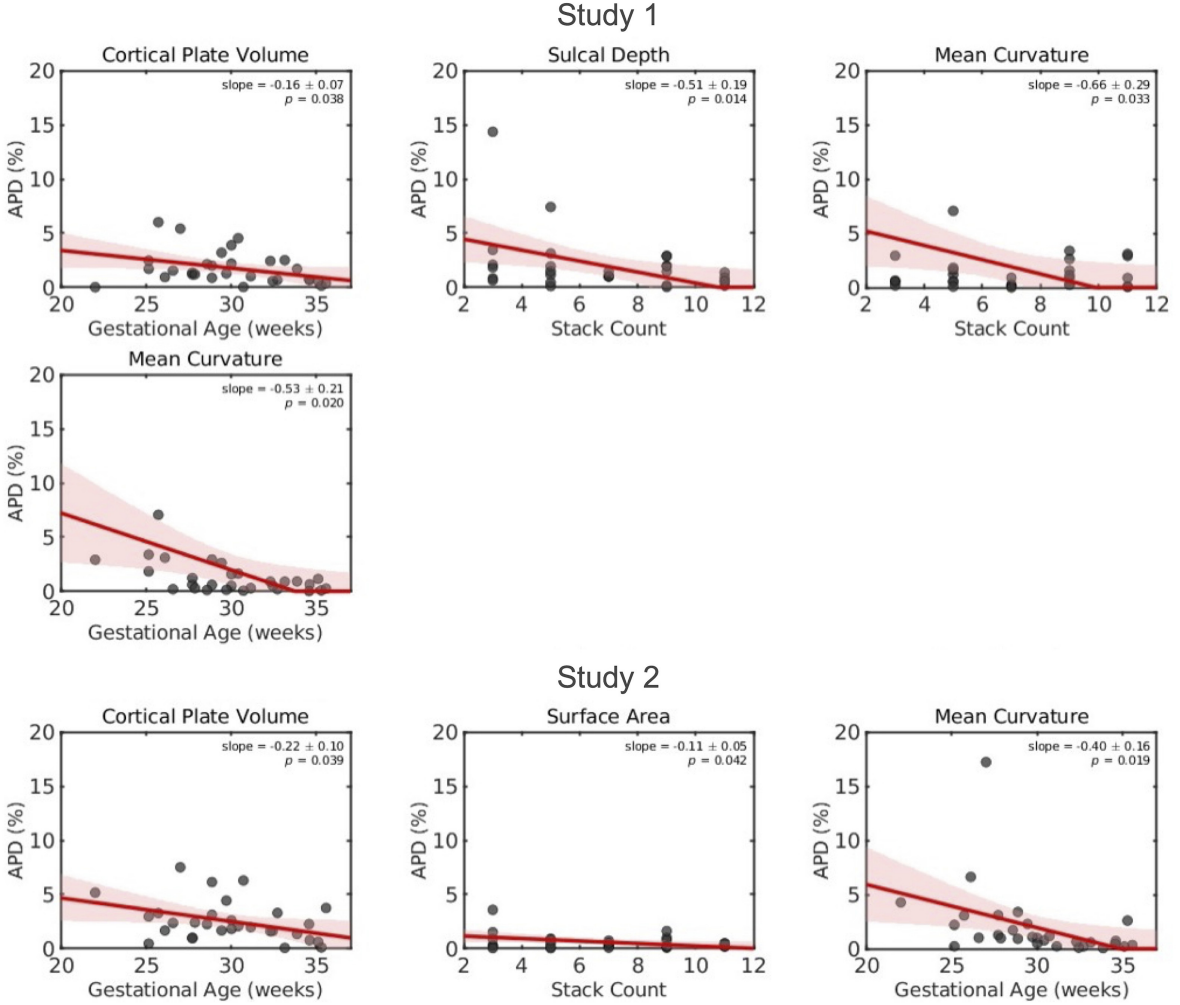
Within-session reliability of NeSVoR-derived cortical metrics by imaging factors and GA. Scatter plots show APD (%) for cortical metrics (y-axis) against stack count or GA (x-axis) for Study 1 (top) and Study 2 (bottom). Points represent individual subjects, regression lines are displayed for covariate–metric pairs with statistically significant associations (pre-FDR *p* < 0.05), and shaded bands indicate the 95% CIs of the regression fits.

Multiple linear regression was used to assess the independent effects of mean stack quality, quality difference, stack count, and GA on measurement variability from NeSVoR reconstructions (Figure 3 and Table 2). Higher mean stack quality was associated with lower APD, reflecting better measurement reliability, for cortical plate volume (β = −3.53 ± 1.76, *p* = 0.056) and mean curvature (β = −10.14 ± 4.98, *p* = 0.053) in Study 1, as well as for mean sulcal depth (β = −4.11 ± 2.09, *p* = 0.061) and mean curvature (β = −8.49 ± 4.18, *p* = 0.053) in Study 2, although none of these trends reached statistical significance (all *p* > 0.05). Quality difference was not significantly associated with APD in any metric. Increasing stack counts was associated with lower APD, with significant effects on mean sulcal depth (β = −0.51 ± 0.19, *p* = 0.014) and mean curvature (β = −0.66 ± 0.29, *p* = 0.033) in Study 1, and surface area (β = −0.11 ± 0.05, *p* = 0.042) in Study 2. Again, none of these associations were significant after FDR correction. Measurement reliability improved with increasing GA for several metrics in both studies. Higher GA was associated with reduced APD for cortical plate volume (Study 1: β = −0.16 ± 0.08, *p* = 0.038; Study 2: β = −0.22 ± 0.10, *p* = 0.039) and mean curvature (Study 1: β = −0.53 ± 0.21, *p* = 0.020; Study 2: β = −0.40 ± 0.16, *p* = 0.019), although these associations again did not remain significant after FDR correction. Bootstrap analysis confirmed the overall stability of the observed regression patterns (Supplementary Table 1). In NeSVoR, mean stack quality, stack count, and GA showed high directional consistency across metrics (typically ∼85–100% in Study 1 and ∼79–100% in Study 2, with some lower values for stack count and surface area), whereas quality difference remained near chance (∼50–56%) across all metrics and studies, consistent with the absence of a stable effect.

**TABLE 2:** Multiple linear regression between APD (NeSVoR-derived) and mean stack quality, quality difference, stack count, and GA.

Study	Biometric measure	Mean stack quality (β ± SE, p)	Quality difference (β ± SE, p)	Stack count (β ± SE, p)	GA (β ± SE, p)
Study 1 (S1–S2)	Cortical plate volume	−3.53 ± 1.76 (0.056)	−6.02 ± 3.51 (0.098)	−0.18 ± 0.10 (0.094)	−0.16 ± 0.08 (0.038*)
	Surface area	−1.74 ± 1.85 (0.355)	−3.70 ± 3.68 (0.324)	−0.18 ± 0.11 (0.113)	−0.09 ± 0.08 (0.247)
	Mean sulcal depth	−4.95 ± 3.28 (0.143)	−2.69 ± 6.51 (0.683)	−0.51 ± 0.19 (0.014*)	−0.22 ± 0.14 (0.132)
	Mean curvature	−10.14 ± 4.98 (0.053)	−5.65 ± 9.91 (0.574)	−0.66 ± 0.29 (0.033*)	−0.53 ± 0.21 (0.020*)
Study 2 (S3–S4)	Cortical plate volume	−1.24 ± 2.62 (0.640)	7.65 ± 4.96 (0.136)	−0.01 ± 0.14 (0.989)	−0.22 ± 0.10 (0.039*)
	Surface area	−1.42 ± 1.00 (0.169)	−0.52 ± 1.90 (0.787)	−0.11 ± 0.05 (0.042*)	−0.01 ± 0.04 (0.863)
	Mean sulcal depth	−4.11 ± 2.09 (0.061)	−2.17 ± 3.97 (0.590)	−0.15 ± 0.11 (0.184)	−0.16 ± 0.08 (0.061)
	Mean curvature	−8.49 ± 4.18 (0.053)	−6.34 ± 4.93 (0.431)	−0.44 ± 0.22 (0.057)	−0.40 ± 0.16 (0.019*)

Regression coefficients (β ± SE, unstandardized) and corresponding *p*-values (pre-FDR, in parentheses) are shown for each covariate. An asterisk (*) denotes statistical significance at uncorrected *p* < 0.05.

Exploratory analyses of slice-thickness variables indicated limited effects on measurement reliability. In NeSVoR reconstructions, only one pre-FDR association was observed (Study 1, cortical plate volume: β = 1.10 ± 0.45, *p* = 0.023); however, this exploratory finding did not remain significant after FDR correction. Furthermore, no other metrics showed significant associations in either Study 1 or Study 2 (Supplementary Table 2).

### Measurement reliability from SVRTK reconstructions

To provide an additional reference, the same within-session reliability framework was applied to SVRTK reconstructions. APD values ranged below 5% across metrics (Supplementary Figure 2A), but were generally higher than those observed in NeSVoR reconstructions (Table 1 and Supplementary Table 3A). Paired *t*-tests comparing APD values (NeSVoR—SVRTK) confirmed this pattern, showing lower APDs for NeSVoR in cortical plate volume in both Study 1 (*t* = −2.55, *p* = 0.034, Cohen’s *d* = 0.70) and Study 2 (*t* = −2.54, *p* = 0.034, Cohen’s *d* = 0.63), surface area in Study 2 (*t* = −3.42, *p* = 0.015, Cohen’s *d* = 0.90), and mean sulcal depth in Study 2 (*t* = −2.81, *p* = 0.034, Cohen’s *d* = 0.71). All *p*-values were FDR-corrected across metrics and studies (Supplementary Table 3B).

In SVRTK, cortical plate volume showed the highest mean APD values (Study 1: 4.07 ± 4.43%; Study 2: 4.84 ± 4.88%). Surface area showed the lowest mean APD (Study 1: 1.81 ± 2.28%; Study 2: 1.84 ± 1.81%). Mean sulcal depth and mean curvature showed intermediate mean APD, ranging from 2.52 to 3.60%. ICC values were also high across metrics (0.976–0.996 in Study 1; 0.984–0.997 in Study 2; Supplementary Figure 2B). RCs indicating within-session reliability in the original measurement units for SVRTK reconstructions were 6.49 cm^3^ [4.54, 12.84] in Study 1 and 8.01 cm^3^ [6.34, 15.09] for cortical plate volume, 1921.0 mm^2^ [1148.0, 3944.1] and 1542.3 mm^2^ [1406.2, 2747.8] for surface area, 0.44 mm [0.36, 0.82] and 0.43 mm [0.34, 0.86] for mean sulcal depth, and 0.03 [0.03, 0.06] and 0.02 [0.02, 0.04] for mean curvature. Across all metrics, RC values were higher than those observed for NeSVoR reconstructions, consistent with APD and ICC-based results (Supplementary Table 3B).

Regression analyses evaluating effects of mean stack quality, quality difference, stack count, and GA revealed patterns that differed from NeSVoR results (Supplementary Figure 3 and Supplementary Table 4). Higher mean stack quality was significantly associated with lower APD for surface area (β = −6.16 ± 2.62, *p* = 0.027) and mean curvature (β = −10.53 ± 4.89, *p* = 0.041) in Study 1, and for mean sulcal depth (β = −10.65 ± 4.45, *p* = 0.024) in Study 2. Trends with mean stack quality were also observed in Study 2 for surface area (β = −4.17 ± 2.08, *p* = 0.056) and mean curvature (β = −6.81 ± 3.43, *p* = 0.058). Quality difference showed no significant association with APD for any metric (all *p* > 0.05). Stack count effects were inconsistent: in Study 2, surface area showed a small positive association with APD (β = +0.24 ± 0.11, *p* = 0.044), whereas other metrics did not. GA was significantly associated with reduced APD for mean curvature (β = −0.55 ± 0.21, *p* = 0.016) in Study 1. None of the associations remained significant after FDR correction. In SVRTK, directional consistency was more variable across predictors and metrics but remained broadly consistent with the main regression results (Supplementary Table 1).

In SVRTK reconstructions, no thickness-related associations were observed for any metric (Supplementary Table 5).

## Discussion

This study evaluated the within-session reliability of automated cortical plate volumetric and surface-based metrics derived from fetal brain MRI using two distinct SVR reconstruction methods. Across all metrics, NeSVoR reconstructions demonstrated consistently high reliability (mean APD < 3%, ICC > 0.99), while SVRTK reconstructions showed similarly high reliability (mean APD < 5%, ICC > 0.97). These findings extend prior fetal MRI reliability studies that primarily focused on linear biometric measures and segmentation overlap (Ciceri et al., 2023; Specktor-Fadida et al., 2024), demonstrating reproducible whole-brain cortical volume and surface measurements under realistic acquisition variability. They are also consistent with findings from a recent multicenter study, which reported that reconstruction-related bias was below the size of a voxel and volumetric differences were < 3% across sites (Sanchez et al., 2024). For context, in fetal ultrasound, the most widely used modality for clinical prenatal assessment, automated measurements of skull biometry have demonstrated good to excellent repeatability, though performance varies by anatomical target (Mlodawski et al., 2024). The present study extends this line of work to 3D cortical morphometric measures derived from fetal MRI, demonstrating that automated reconstruction and analysis pipelines can yield robust quantitative measures that may complement established ultrasound-based fetal assessment.

A key feature of this work is the use of disjoint subsets of stacks acquired within a single imaging session. This design was intentionally chosen to isolate reconstruction- and acquisition-related variability from true biological change. In fetal neuroimaging, classical test-retest paradigms are difficult to interpret because even short inter-scan intervals can introduce substantial developmental changes in cortical volume, surface area, and folding, particularly during mid-gestation. While repeated scans within the same session do not capture temporal reproducibility, we believe they represent a methodologically meaningful approach for assessing internal consistency of automated pipelines in the fetal setting. Consequently, the reliability estimates reported here should be interpreted as measures of technical reproducibility rather than longitudinal stability.

### Influence of imaging factors and gestational age

Among the evaluated variables, stack count emerged as the most consistent imaging-related factor influencing measurement stability, with variability generally decreasing as more stacks were included in the reconstruction. This effect was most evident for curvature- and depth-based measures, which depend on local surface geometry and are therefore likely more sensitive to subtle reconstruction or segmentation variability. Mean stack quality showed weaker associations with reliability once a minimum quality threshold was met, suggesting that beyond excluding severely corrupted stacks, increasing data quantity may be more beneficial than modest improvements in average quality. Notably, differences in mean quality between paired subsets did not meaningfully influence reliability, supporting the robustness of automated morphometry under moderate heterogeneity in stack quality within a session. Given the modest sample size and the number of covariates, these regression analyses should be considered exploratory and interpreted with caution. Nevertheless, bootstrap resampling demonstrated that the observed associations are directionally stable across resamples, supporting the robustness of the reported trends.

GA was associated with improved reliability for several metrics in NeSVoR reconstructions. This likely reflects a combination of reduced fetal motion at later gestation, increased brain size yielding higher effective spatial resolution, and the fact that segmentation-related variations constitute a smaller fraction of total cortical volume in larger brains. These effects occurred despite increasing cortical complexity at higher GA, which generally poses greater challenges for surface reconstruction. Importantly, because this study assessed within-session consistency, systematic biases that remain stable across subsets could still yield high reliability. Thus, improved reliability at later gestation does not necessarily imply greater biological accuracy, underscoring the need to distinguish reproducibility from validity.

Metric-specific sensitivity was also observed. Surface area exhibited the greatest stability across imaging conditions, whereas mean curvature showed the strongest dependence on stack count and GA. Cortical plate volume and sulcal depth were intermediate, likely due to their reliance on accurate delineation of the cortical boundary making them more susceptible to segmentation errors arising from acquisition-related reconstruction artifacts. Additional acquisition parameters such as slice thickness, contrast, and overall signal-to-noise ratio are known to affect both SVR and surface reconstruction. In this dataset, slice thickness varied within a relatively narrow range (2–4 mm), and exploratory analyses suggested only a limited secondary influence on reliability. Broader acquisition variability may reveal stronger effects and should be further investigated, particularly in larger and more heterogeneous samples to establish clinical applicability.

### Comparison of reconstruction methods and methodological scope

Although NeSVoR and SVRTK produced broadly similar reliability profiles, NeSVoR consistently yielded lower APD and higher ICC across metrics. These differences likely reflect underlying differences in their methodological approach. NeSVoR models the reconstructed volume as a continuous implicit function and explicitly estimates slice-level noise and acquisition uncertainty (Xu et al., 2023), which may allow it to more effectively accommodate heterogeneous stack quality and benefit from increased data quantity. In contrast, SVRTK relies on iterative rigid alignment and robust outlier handling within a super-resolution framework (Uus et al., 2020; Xu et al., 2023), which may be more sensitive to average image quality. GA had minimal influence on SVRTK-derived reliability, while NeSVoR showed higher reliability at later gestations. The methodological features of NeSVoR, such as explicit modeling of slice acquisition and noise variance, may have a greater positive effect on the reliability in late gestation, benefiting from larger brain size, higher effective resolution, and reduced fetal motion. However, such effects observed exclusively with NeSVoR should be further investigated in the future, ideally in larger samples to assess statistical robustness.

It is important to note that only two SVR methods were evaluated here, representing two widely used but distinct classes of fetal reconstruction approaches. Other methods, including NiftyMIC (Ebner et al., 2020), MIALSSRTK (Tourbier et al., 2020), deformable SVR techniques (Uus et al., 2020), and other neural network-based approaches (Wu et al., 2025), may exhibit different reliability characteristics. Consequently, the present results should be interpreted as comparative benchmarks rather than an exhaustive evaluation of all available reconstruction strategies.

Additionally, all cortical surfaces in this study were generated using a single automated segmentation and surface reconstruction pipeline. As a result, the reported reliability reflects the combined performance of reconstruction, segmentation, and surface extraction. While our surface extraction approach enforces topological constraints, explicit quantification of surface integrity or topological defects was beyond the scope of this study. Future work could build on these benchmarks to explore alternative segmentation architectures, training datasets, or topology enforcement strategies, which may further enhance reproducibility, particularly for datasets that differ in appearance from those used during model training.

From a biological perspective, the observed levels of technical variability are small relative to the magnitude of GA-related changes reported for cortical plate volume and surface metrics in prior studies (Scott et al., 2011). This suggests that, under typical acquisition conditions, automated fetal morphometry is unlikely to obscure large-scale developmental effects. However, this study does not establish sensitivity to subtle pathological deviations, nor does it evaluate diagnostic performance. Therefore, while the results provide essential quantitative benchmarks, their generalizability to atypically developing populations remains to be assessed.

### Practical implications and future directions

These findings demonstrate that automated fetal MRI pipelines can achieve high within-session reliability even under suboptimal but realistic acquisition conditions, including low stack count and moderate motion-related degradation. Increasing the number of usable stacks provides the most consistent gains in reliability, while surface area emerges as the most stable metric across reconstruction methods. At the same time, subtle method-specific sensitivities highlight that imaging factors and algorithmic design interact to shape measurement stability. While stack count emerges as the important factor, continued development of acquisition protocols, including improvements in motion mitigation, faster imaging, and motion-robust reconstruction strategies, will be important for further enhancing data quality and extending reliability across broader clinical and research scenarios (Zhang et al., 2024; Zhang et al., 2020).

Future work should extend this framework to multi-site datasets, broader acquisition parameter ranges, including different scanners and acquisition protocols, as well as evaluate additional reconstruction and segmentation approaches. Importantly, while in our work we restricted the cohort to typically developing fetuses to minimize biological variability related to atypical cortical development, future studies will need to determine whether these findings generalize to pathological populations. Larger samples and more heterogeneous datasets will also allow rigorous assessment of covariate effects and improve generalizability. Such efforts will be essential for translating technical reproducibility into biological interpretability and, ultimately, clinical utility.

While the reported reliability reflects within-session repeatability within a single MRI examination acquired on one scanner using a fixed protocol, our within-session design provides a practical framework for fetal imaging. This study therefore establishes initial quantitative benchmarks under realistic fetal MRI constraints and provides a reference point for methodological robustness, while clearly distinguishing internal consistency from biological validity.

## Data Availability

Image-processing and analysis scripts are available via the lab’s centralized GitHub (HYPERLINK https://github.com/FNNDSC). Anonymized MRI data, associated metadata, and derivative files used in this study may be obtained from the corresponding author (kiho.im@childrens.harvard.edu) upon reasonable request and execution of a data-use agreement, as required by institutional policies.
