# Supplementary material for: Evaluating reliability of automated quantitative brain morphometry from fetal T2-weighted MRI
Source: Front Neurosci. 2026 Jun 1;20:1817732. doi: 10.3389/fnins.2026.1817732 (PMC13265515; doi:10.3389/fnins.2026.1817732)
Supplement: Supplementary file 1 [file Data_Sheet_1.docx]

**Supplementary Figures**


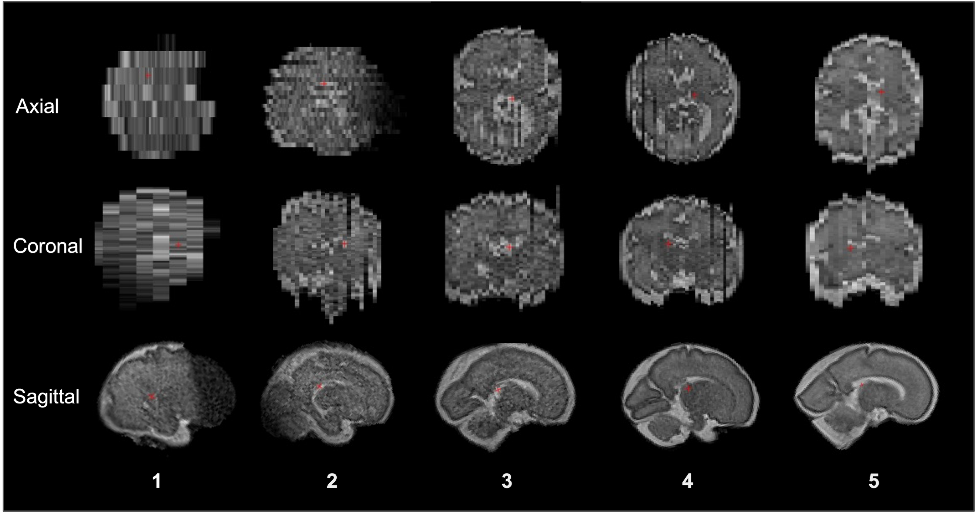


**Supplementary Figure 1.** **Representative fetal brain MRI slices illustrating the HASTE stack five-point quality scale (from 1: unusable to 5: excellent quality).**


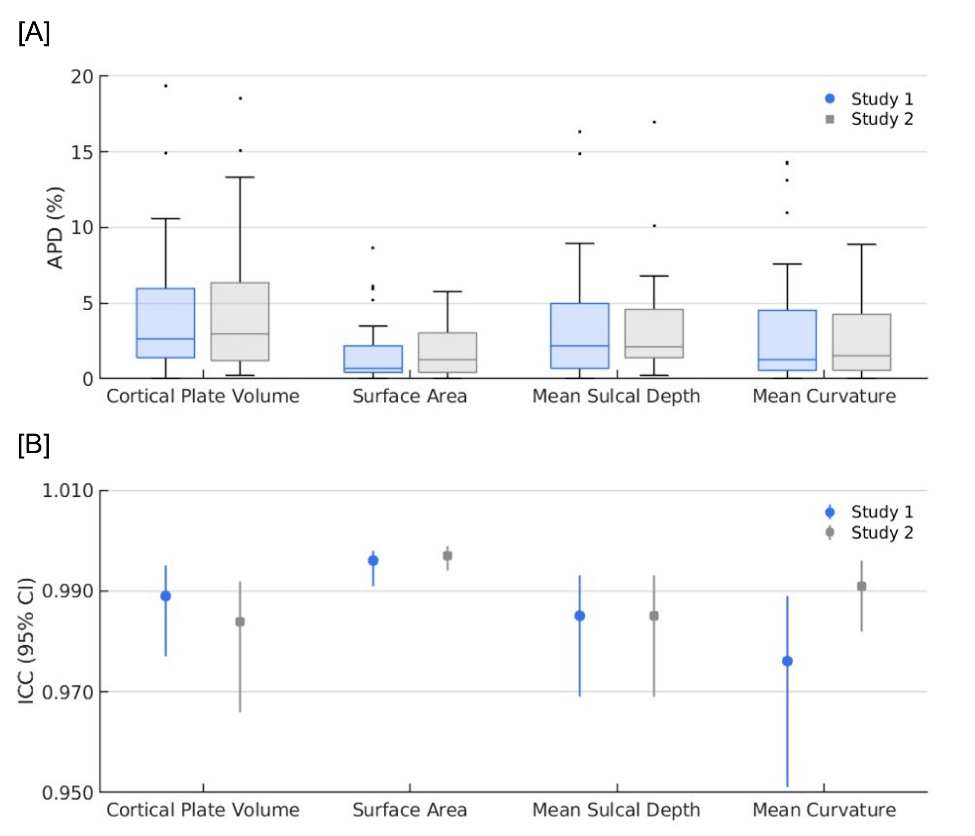


**Supplementary Figure 2. Within-session reliability of SVRTK-derived cortical metrics.**

(A) Box plots display APD (%) across subjects for cortical plate volume, surface area, mean sulcal depth, and mean curvature in Study 1 and Study 2**.**

(B) ICC(2,1) estimates with error bars indicating 95% CIs for cortical plate volume, surface area, mean sulcal depth, and mean curvature in Study 1 and Study 2 (dashed line at ICC = 1).


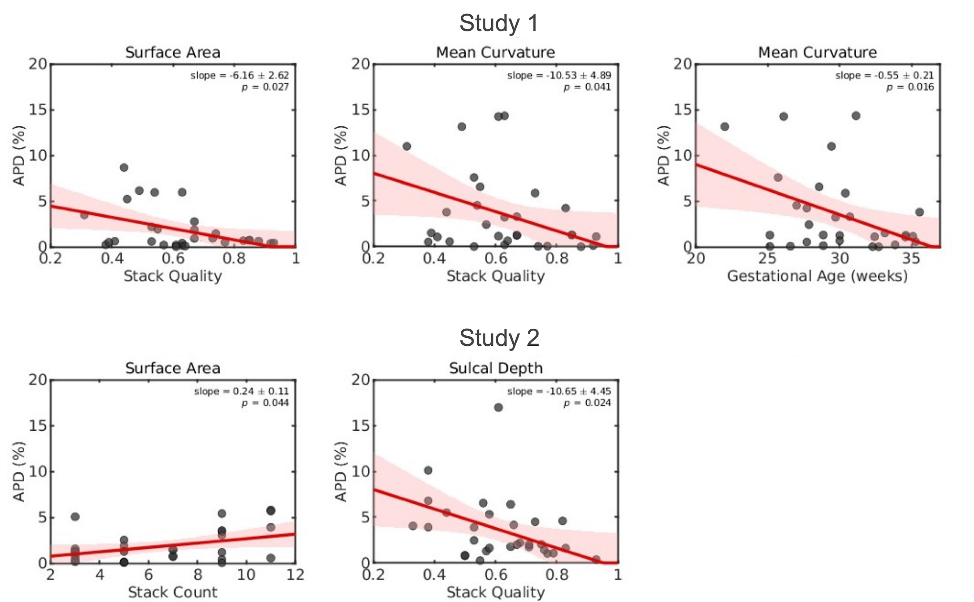


**Supplementary Figure 3. Within-session reliability of SVRTK-based cortical metrics with imaging factors and GA.**
Scatter plots show APD (%) for cortical metrics (y-axis**) against mean stack quality, stack count or GA (x-axis)** for **Study 1 (top)** and **Study 2 (bottom).** Points represent individual subjects, regression lines are displayed for covariate–metric pairs with statistically significant associations (pre-FDR p < 0.05), and shaded bands indicate the 95% CIs of the regression fits.


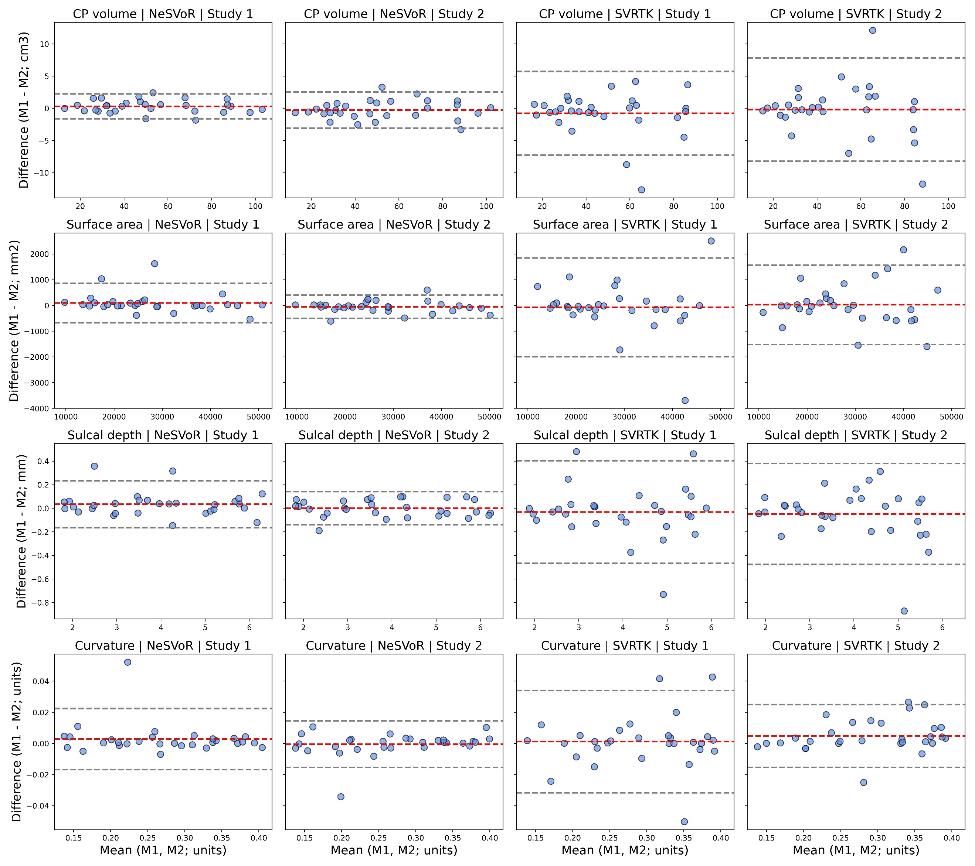


**Supplementary Figure 4. Bland–Altman analysis summarizing absolute measure agreement.**

Plots show agreement between paired reconstructions across cortical metrics (rows: cortical plate volume, surface area, mean sulcal depth, mean curvature). Columns represent reconstruction method (NeSVoR, SVRTK) and within-session split (Study 1 and Study 2). The solid red line indicates mean bias; dashed lines indicate ±1.96 standard deviations (limits of agreement). Axes are consistent within measures to enable direct visual comparison.

**Supplementary Tables**

#### **Supplementary Table 1.** Bootstrap stability of multiple linear regression coefficients for APD (NeSVoR and SVRTK reconstructions, Study 1 and Study 2). Values indicate directional consistency (% of bootstrap iterations retaining the same sign as the full-sample estimate) across 1000 subject-level resamples for each predictor and measure.

| \| **NeSVoR** \| \| \| \| \| \| \| --- \| --- \| --- \| --- \| --- \| --- \| \| Study \| Measure \| Mean Stack Quality  (%) \| Quality Difference  (%) \| Stack Count  (%) \| GA  (%) \| \| Study 1  (S1–S2) \| Cortical Plate Volume \| 93 \| 52 \| 92 \| 97 \| \|  \| Surface Area \| 85 \| 56 \| 88 \| 91 \| \|  \| Mean Sulcal Depth \| 89 \| 51 \| 99 \| 95 \| \|  \| Mean Curvature \| 96 \| 50 \| 92 \| 100 \| \| Study 2  (S3–S4) \| Cortical Plate Volume \| 64 \| 51 \| 54 \| 93 \| \|  \| Surface Area \| 93 \| 54 \| 96 \| 58 \| \|  \| Mean Sulcal Depth \| 99 \| 52 \| 79 \| 98 \| \|  \| Mean Curvature \| 99 \| 52 \| 83 \| 100 \| | \| **SVRTK** \| \| \| \| \| \| \| --- \| --- \| --- \| --- \| --- \| --- \| \| Study \| Measure \| Mean Stack Quality  (%) \| Quality Difference  (%) \| Stack Count  (%) \| GA  (%) \| \| Study 1  (S1–S2) \| Cortical Plate Volume \| 90 \| 51 \| 98 \| 70 \| \|  \| Surface Area \| 98 \| 55 \| 67 \| 50 \| \|  \| Mean Sulcal Depth \| 97 \| 51 \| 77 \| 77 \| \|  \| Mean Curvature \| 99 \| 96 \| 82 \| 87 \| \| Study 2  (S3–S4) \| Cortical Plate Volume \| 80 \| 52 \| 90 \| 79 \| \|  \| Surface Area \| 72 \| 51 \| 100 \| 98 \| \|  \| Mean Sulcal Depth \| 79 \| 51 \| 72 \| 58 \| \|  \| Mean Curvature \| 89 \| 52 \| 98 \| 74 \| |
| --- | --- | --- | --- | --- | --- | --- | --- | --- | --- | --- | --- | --- | --- | --- | --- | --- | --- | --- | --- | --- | --- | --- | --- | --- | --- | --- | --- | --- | --- | --- | --- | --- | --- | --- | --- | --- | --- | --- | --- | --- | --- | --- | --- | --- | --- | --- | --- | --- | --- | --- | --- | --- | --- | --- | --- | --- | --- | --- | --- | --- | --- | --- | --- | --- | --- | --- | --- | --- | --- | --- | --- | --- | --- | --- | --- | --- | --- | --- | --- | --- | --- | --- | --- | --- | --- | --- | --- | --- | --- | --- | --- | --- | --- | --- | --- | --- | --- | --- | --- | --- | --- | --- | --- | --- | --- | --- | --- | --- | --- | --- | --- | --- | --- | --- | --- | --- | --- | --- | --- | --- | --- |

#### **Supplementary Table 2. Multiple linear regression results of APD (NeSVoR) with mean stack quality, quality difference, mean slice thickness, thickness difference, stack count, and GA.**

#### Regression coefficients (β ± SE, unstandardized) and corresponding p-values (pre-FDR, in parentheses) are shown for each covariate. An asterisk (*) denotes statistical significance at uncorrected p < 0.05.

| Study | Biometric Measure | Mean Stack Quality  (β ± SE, p) | Quality Difference  (β ± SE, p) | Mean Slice Thickness  (β ± SE, p) | Thickness Difference  (β ± SE, p) | Stack Count  (β ± SE, p) | GA  (β ± SE, p) |
| --- | --- | --- | --- | --- | --- | --- | --- |
| Study 1 (S1–S2) | Cortical Plate Volume | -3.60 ± 1.65 (0.040*) | 5.34 ± 3.25 (0.114) | 1.10 ± 0.45 (0.023*) | 0.42 ± 0.82 (0.617) | -0.23 ± 0.10 (0.034*) | -0.21 ± 0.07 (0.008*) |
|  | Surface Area | -1.91 ± 1.90 (0.324) | -3.93 ± 3.74 (0.304) | 0.64 ± 0.52 (0.231) | -0.16 ± 0.95 (0.866) | -0.19 ± 0.12 (0.115) | -0.11 ± 0.08 (0.201) |
|  | Mean Sulcal Depth | -5.13 ± 3.34 (0.139) | -3.29 ± 6.59 (0.623) | 1.19 ± 0.92 (0.208) | 0.12 ± 1.67 (0.945) | -0.55 ± 0.20 (0.013*) | -0.26 ± 0.15 (0.093) |
|  | Mean Curvature | -10.64 ± 5.11 (0.049*) | -5.11 ± 10.08 (0.617) | 1.70 ± 1.40 (0.236) | -0.60 ± 2.56 (0.815) | -0.68 ± 0.31 (0.039*) | -0.57 ± 0.23 (0.019*) |
| Study 2 (S3–S4) | Cortical Plate Volume | -0.68 ± 2.81 (0.811) | 6.70 ± 5.57 (0.241) | -0.37 ± 0.81 (0.653) | 0.56 ± 1.05 (0.600) | -0.01 ± 0.14 (0.945) | -0.21 ± 0.11 (0.068) |
|  | Surface Area | -1.48 ± 1.08 (0.183) | 0.01 ± 2.13 (0.996) | 0.19 ± 0.31 (0.540) | 0.05 ± 0.40 (0.895) | -0.12 ± 0.06 (0.048*) | -0.02 ± 0.04 (0.707) |
|  | Mean Sulcal Depth | -3.82 ± 2.26 (0.105) | -2.57 ± 4.49 (0.573) | -0.16 ± 0.65 (0.811) | 0.32 ± 0.85 (0.710) | -0.16 ± 0.65 (0.811) | -0.16 ± 0.09 (0.095) |
|  | Mean Curvature | -9.49 ± 4.48 (0.045*) | -5.05 ± 8.88 (0.575) | 0.52 ± 1.29 (0.693) | -1.10 ± 1.67 (0.516) | -0.46 ± 0.23 (0.058) | -0.39 ± 0.18 (0.038*) |

#### **Supplementary Table 3. Within-session reliability of SVRTK-derived quantitative cortical measurements.**

#### **A.** Absolute percent difference (APD, mean ± SD), intraclass correlation coefficients [ICC(2,1), 95% CI], and repeatability coefficients [RC, 95% CI] for cortical plate volume, surface area, mean sulcal depth, and mean curvature in Study 1 (S1–S2) and Study 2 (S3–S4). RC values are reported in cm³ for cortical plate volume, mm² for surface area, mm for mean sulcal depth, and curvature units for mean curvature.

**B.** Paired statistical comparisons of absolute percent difference (APD) between NeSVoR and SVRTK reconstructions across cortical metrics and studies. Reported are t-statistics from paired t-tests, corresponding false discovery rate (FDR)-corrected p-values, and effect sizes (Cohen’s d). Negative t-values indicate lower APD for NeSVoR relative to SVRTK.

**A.**

| Biometric Measure | Study 1 | | | Study 2 | | |
| --- | --- | --- | --- | --- | --- | --- |
|  | Mean APD (%) ± SD | ICC [95% CI] | RC [95% CI] | Mean APD (%) ± SD | ICC [95% CI] | RC [95% CI] |
| Cortical Plate Volume | 4.07 ± 4.43 | 0.989 [0.977, 0.995] | 6.49 [4.54, 12.84] | 4.84 ± 4.88 | 0.984 [0.966, 0.992] | 8.01 [6.34, 15.09] |
| Surface Area | 1.81 ± 2.28 | 0.996 [0.991, 0.998] | 1921.0 [1148.0, 3944.1] | 1.84 ± 1.81 | 0.997 [0.994, 0.999] | 1542.3 [1406.2, 2747.8] |
| Mean Sulcal Depth | 3.60 ± 4.15 | 0.985 [0.969, 0.993] | 0.44  [0.36, 0.82] | 3.58 ± 3.44 | 0.985 [0.969, 0.993] | 0.43  [0.34, 0.86] |
| Mean Curvature | 3.54 ± 4.37 | 0.976 [0.951, 0.989] | 0.03  [0.03, 0.06] | 2.52 ± 2.73 | 0.991 [0.982, 0.996] | 0.02  [0.02, 0.04] |

**B.**

|  |  | **T** | **p (corr.)** | **Cohen-D** |
| --- | --- | --- | --- | --- |
| Cortical Plate Volume | *Study 1* | -2.55 | 0.034 | 0.70 |
|  | *Study 2* | -2.54 | 0.034 | 0.63 |
| Surface Area | *Study 1* | -2.26 | 0.051 | 0.52 |
|  | *Study 2* | -3.42 | 0.015 | 0.90 |
| Mean Sulcal Depth | *Study 1* | -1.95 | 0.081 | 0.46 |
|  | *Study 2* | -2.81 | 0.034 | 0.71 |
| Mean Curvature | *Study 1* | -1.50 | 0.165 | 0.35 |
|  | *Study 2* | -0.79 | 0.438 | 0.19 |

.

#### **Supplementary Table 4. Multiple linear regression results of APD (SVRTK) with mean stack quality, quality difference, stack count, and GA.**

#### Regression coefficients (β ± SE, unstandardized) and corresponding p-values (pre-FDR, in parentheses) are shown for each covariate. An asterisk (*) denotes statistical significance at uncorrected p < 0.05.

| Study | Biometric Measure | Mean Stack Quality  (β ± SE, p) | Quality Difference  (β ± SE, p) | Stack Count  (β ± SE, p) | GA  (β ± SE, p) |
| --- | --- | --- | --- | --- | --- |
| Study 1 (S1–S2) | Cortical Plate Volume | -5.12 ± 5.39 (0.351) | -11.69 ± 11.12 (0.303) | 0.24 ± 0.31 (0.457) | -0.11 ± 0.23 (0.650) |
|  | Surface Area | -6.16 ± 2.62 (0.027*) | -6.42 ± 5.40 (0.245) | -0.22 ± 0.15 (0.166) | 0.01 ± 0.11 (0.979) |
|  | Mean Sulcal Depth | -8.52 ± 4.77 (0.086) | -17.94 ± 9.84 (0.080) | -0.21 ± 0.28 (0.462) | -0.19 ± 0.21 (0.376) |
|  | Mean Curvature | -10.53 ± 4.89 (0.041*) | 5.47 ± 10.09 (0.593) | -0.09 ± 0.28 (0.750) | -0.55 ± 0.21 (0.016*) |
| Study 2 (S3–S4) | Cortical Plate Volume | -6.76 ± 6.02 (0.272) | -6.77 ± 12.48 (0.592) | 0.61 ± 0.33 (0.074) | 0.21 ± 0.25 (0.401) |
|  | Surface Area | -4.17 ± 2.08 (0.056) | 5.47 ± 4.32 (0.217) | 0.24 ± 0.11 (0.044*) | 0.06 ± 0.09 (0.458) |
|  | Mean Sulcal Depth | -10.65 ± 4.45 (0.024*) | 1.61 ± 9.23 (0.863) | -0.41 ± 0.24 (0.100) | -0.01 ± 0.18 (0.978) |
|  | Mean Curvature | -6.81 ± 3.43 (0.058) | 2.02 ± 7.12 (0.779) | 0.22 ± 0.19 (0.254) | -0.15 ± 0.14 (0.290) |

#### **Supplementary Table 5. Multiple linear regression results of APD (SVRTK) with mean stack quality, quality difference, mean slice thickness, thickness difference, stack count, and GA.**

#### Regression coefficients (β ± SE, unstandardized) and corresponding p-values (pre-FDR, in parentheses) are shown for each covariate. An asterisk (*) denotes statistical significance at uncorrected p < 0.05.

| Study | Biometric Measure | Mean Stack Quality  (β ± SE, p) | Quality Difference  (β ± SE, p) | Mean Slice Thickness  (β ± SE, p) | Thickness Difference  (β ± SE, p) | Stack Count  (β ± SE, p) | GA  (β ± SE, p) |
| --- | --- | --- | --- | --- | --- | --- | --- |
| Study 1  (S1–S2) | Cortical Plate Volume | -3.58 ± 5.33 (0.508) | -11.20 ± 10.82 (0.311) | -2.79 ± 1.54 (0.082) | 2.05 ± 2.78 (0.469) | 0.30 ± 0.33 (0.385) | 0.15 ± 0.24 (0.529) |
|  | Surface Area | -5.72 ± 2.64 (0.041*) | -5.96 ± 5.35 (0.277) | -1.25 ± 0.76 (0.114) | 0.22 ± 1.38 (0.873) | -0.16 ± 0.16 (0.335) | 0.04 ± 0.12 (0.762) |
|  | Mean Sulcal Depth | -8.47 ± 4.91 (0.098) | -16.81 ± 9.97 (0.105) | -1.65 ± 1.42 (0.255) | -1.20 ± 2.56 (0.644) | -0.07 ± 0.31 (0.815) | -0.11 ± 0.22 (0.604) |
|  | Mean Curvature | -10.75 ± 5.14 (0.048*) | 4.86 ± 10.44 (0.646) | 1.15 ± 1.38 (0.448) | 0.32 ± 2.68 (0.905) | -0.16 ± 0.32 (0.615) | -0.59 ± 0.23 (0.017*) |
| Study 2  (S3–S4) | Cortical Plate Volume | -8.91 ± 6.32 (0.172) | -4.44 ± 13.54 (0.746) | 0.93 ± 1.98 (0.642) | -2.88 ± 2.54 (0.269) | 0.60 ± 0.33 (0.086) | 0.24 ± 0.27 (0.378) |
|  | Surface Area | -4.73 ± 2.21 (0.043*) | 5.65 ± 4.74 (0.245) | 0.08 ± 0.69 (0.911) | -0.91 ± 0.89 (0.314) | 0.24 ± 0.11 (0.044*) | 0.08 ± 0.09 (0.385) |
|  | Mean Sulcal Depth | -10.41 ± 4.79 (0.040*) | -0.57 ± 10.28 (0.957) | -0.85 ± 1.50 (0.576) | -0.48 ± 1.93 (0.805) | -0.39 ± 0.25 (0.140) | -0.04 ± 0.20 (0.840) |
|  | Mean Curvature | -6.60 ± 3.72 (0.089) | 1.40 ± 7.98 (0.862) | -0.24 ± 1.17 (0.837) | 0.11 ± 1.50 (0.945) | 0.22 ± 0.20 (0.268) | -0.14 ± 0.16 (0.374) |
